# A novel *BRCA1* mutation in a patient with breast and ovarian cancer: A case report

**DOI:** 10.3892/ol.2013.1440

**Published:** 2013-07-03

**Authors:** JOSEFA SALGADO, MARTA SANTISTEBAN, CRISTINA GUTIÉRREZ, CARMEN GIL, MAITANE ROBLES, ADRIANA VIEDMA, ANA PATIÑO-GARCÍA

**Affiliations:** 1 Clinical Genetics Unit, University Clinic of Navarra (CUN), Pamplona, Navarra 31008, Spain; 2 Department of Oncology, University Clinic of Navarra (CUN), Pamplona, Navarra 31008, Spain

**Keywords:** BRCA1, novel mutation, breast and ovarian cancer

## Abstract

Germline mutations in the human breast cancer genes *BRCA1* and *BRCA2* account for a substantial proportion of familial, early-onset breast and ovarian cancers. The present study reports a novel disease-causing *BRCA1* mutation, nucleotide 3020insCT/c.2901insCT, in a 55-year-old Spanish female with breast and ovarian cancer. This frameshift mutation creates a premature stop codon at amino acid 1000, leading to a truncated BRCA1 protein. To the best of our knowledge, this mutation has not been previously described in the Breast Cancer Information Core (BIC) database or the published literature.

## Introduction

Hereditary breast and ovarian cancer (HBOC) is an autosomal dominant syndrome with incomplete penetrance. The two most commonly mutated genes in HBOC are *BRCA1* and *BRCA2*, which are essential components of the double-strand break repair system (1). Almost 3,500 cancer-associated mutations, scattered throughout the two genes, have so far been reported in the Breast Cancer Information Core (BIC) database (http://research.nhgri.nih.gov/bic/). The present study reports a new germline nucleotide 3020insCT/c.2901insCT mutation detected in the *BRCA1* gene. In general, germline mutations in known breast cancer risk genes account for ~20% of breast cancers associated with a family history. It is therefore crucial to identify these individuals to offer appropriate cancer management and understand the contribution of *BRCA1* and *BRCA2* mutation-associated risks.

## Case report

**Figure 1:**
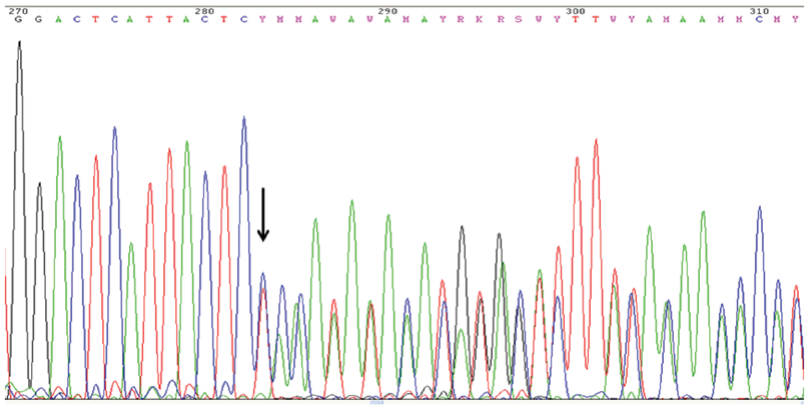
Chromatogram of breast cancer (*BRCA*)*1* exon 11 showing the 3020insCT/c.2901insCT in the heterozygous state (arrow) in the peripheral blood of the patient.

A 55-year-old non-Ashkenazi Spanish female diagnosed with breast cancer (at 51 years old) and ovarian cancer (at 55 years old) and treated at the University Clinic of Navarra (CUN; Pamplona, Navarra, Spain), was transferred to the genetic counseling unit. The clinical history of the patient lead us to consider the possibility of HBOC syndrome. Following verbal and written informed consent, genomic DNA was extracted from a peripheral blood sample and the *BRCA1* and *BRCA2* genes were sequenced on an automated analyzer (ABI PRISM^®^ 3130XL; Applied Biosystems, Foster City, CA, USA). The results were compared to the consensus wild-type sequences (Genbank NM_007294.2 for *BRCA1* and Genbank NM_000059.1 for *BRCA2*). A 3020insCT/c.2901insCT frameshift mutation was identified in exon 11 of *BRCA1* (Fig. 1). The insertion was confirmed by repeated analyses including reverse-primer sequencing. A *BRCA1*-Multiplex Ligation-dependent Probe Amplification (MLPA) analysis was performed, in order to investigate whether the mutation was able to lead to an exon rearrangement. The results indicated that none of the *BRCA1* alleles showed deletion and/or duplication (results not shown).

Genetic analysis was recommended to the only other individual at risk in patient’s family, namely the twin sister, and the analysis showed that she did not carry the mutation.

## Discussion

A 3020insCT/c.2901insCT frameshift mutation in exon 11 of the *BRCA1*, which has yet to be reported in the BIC database, was detected in a 55-year-old non-Ashkenazi Spanish female diagnosed with breast and ovarian cancer. Since other family members were not available for genetic analysis, the segregation of the mutation could not be established. From the literature available, it may be deduced that the mutation leads to the deletion of the coiled-coil domain and BRCA1 C terminus (BRCT) domains of the BRCA1 protein. The coiled-coil region is critical for transcriptional activation through its interaction with the basic leucine zipper (bZIP) domain of the JunB protein. *In vitro* and *in vivo* experiments suggest that this BRCA1-JunB interaction is particularly important for the suppression of ovarian cancer (2). The lack of the coiled-coil domain in the present patient may have been closely correlated with the development of the ovarian cancer. However, BRCA1 has a pivotal function within the BRCA1-associated genome surveillance complex through the coordination of the actions of damage-sensing and executive repair proteins. Solyom *et al*(3) showed that the Abraxas protein serves as a central organizer of a large BRCA1 holoenzyme complex. Abraxas directly binds, via its phosphorylated C terminus, to the BRCA1 BRCT motifs, linking BRCA1 to a core protein complex dedicated to ubiquitin chain recognition and hydrolysis at DNA double-strand breaks (3,4). Moreover, BRCT domains in BRCA1 are able to bind DNA strand breaks and ends *in vitro*, which is enhanced by the formation of the BRCA1-BARD1 heterodimer (5). The structural studies of Kobayashi *et al* showed that the BRCT domain partially inserts into the major groove and makes extensive contacts with the DNA backbone (6), suggesting the possibility that proteins with BRCT domains may act as DNA sensors and transducers of DNA damage response signaling. The mutation identified in the present study would markedly compromise these functions, with profound biological consequences. The premature stop codon at amino acid 1000 leads to a truncated protein that has 70% of its normal length. The advantage of having mutant *BRCA1* human breast cancer cell lines is that the impact of pathogenic human mutations may be evaluated in the context of a human genetic background. A previous study of 41 human breast cancer cell lines identified a *BRCA1* mutant cell line, SUM149PT, with a nucleotide deletion at position 2288 (7). The resulting truncated BRCA1 protein lacked the C-terminal BRCT and coiled-coil domains similar to the present patient. Nuclear BRCA1 protein expression was not detectable in the cell line, therefore corroborating the tumor suppressor function of BRCA1 and the pathogenicity of the mutation.

The next step was to search for bibliographic evidence of the present mutation in *BRCA1-*knockout animal models. The homozygous loss of *BRCA1* generally leads to early embryonic lethality, although it is possible to extend the viability though the removal of p53 function. Among the range of models available, McCarthy *et al* designed truncated human *BRCA1*f22-24/p53^+/−^ mice (harboring the second BRCT domain), that develop estrogen receptor-negative (ER^−^) and progesterone receptor-negative (PR^−^) tumors lacking HER2 protein overexpression and gene amplification (8). This phenotype is similar to 64–90% of human *BRCA1*-mutation breast cancers, so called ‘triple negative’ breast cancers. The immunophenotypic features of the present patient’s tumor indicated a noticeably different pattern, being ER^+^, PR^+^ and ErbB2-negative. It has been reported that 10–36% of *BRCA1* mutation-related invasive breast cancers are, in fact, ER^+^. Furthermore, *BRCA1* mutation carriers who are older or post-menopausal at the time of the diagnosis of breast cancer are more likely to have an ER^+^ breast cancer (9,10). With regard to the origin of these ER^+^*BRCA1*-related breast cancers, Lim *et al* observed the expansion of a committed luminal progenitor population, containing ER^+^ and ER^−^ cells, in preneoplastic tissues of *BRCA1* mutation carriers and proposed the luminal progenitor cells as the cell of origin for *BRCA1*-associated cancers (11). In mouse models with the deletion of *BRCA1*, the expression of ER in the resulting tumors appears to depend on whether *BRCA1* is deleted at an earlier or later stage of cell differentiation (12–14). These studies suggest that *BRCA1*-deficient ER^+^ tumors may derive from *BRCA1* loss in an ER^+^ luminal progenitor cell.

Another key point is the therapeutic approach for ER^+^*BRCA1*-associated breast cancers. Given the availability of effective therapies that exploit defects in homologous recombination, such as PARP-1 inhibitors and cisplatin, it is increasingly important to determine whether these therapies are likely to be effective in ER^+^*BRCA1*-mutant cancers. A recent study by Kaplan *et al* indicated that ER^+^*BRCA1*-related breast cancers are indistinguishable from ER^−^*BRCA1*-related cancers in their nuclear expression of PARP-1, suggesting that ER^+^*BRCA1*-related breast cancers may respond well to drugs that exploit BRCA1 deficiency (15). ER^+^*BRCA1*-related breast cancers appear to be a unique group and efforts should be made to identify the individuals for whom estrogen-modifying agents are likely to be particularly effective.
